# *In vitro* and *in vivo* Repair Effects of the NCF-Col-NHA Aerogel Scaffold Loaded With SOST Monoclonal Antibody and SDF-1 in Steroid-Induced Osteonecrosis

**DOI:** 10.3389/fbioe.2022.825231

**Published:** 2022-03-08

**Authors:** Bing Xu, Zeyu Luo, Duan Wang, Zeyu Huang, Zongke Zhou, Haoyang Wang

**Affiliations:** ^1^ Department of Orthopaedic, West China Hospital, Orthopedic Research Institute, Sichuan University, ChengDu, China; ^2^ Department of Orthopaedic Surgery, Chengdu Second People’s Hospital, ChengDu, China

**Keywords:** aerogel, steroid-induced osteonecrosis, bone repair, sost, bone regenaration

## Abstract

In the current study, we synthesized nanocellulose (NCF)-collagen (Col)-nano hydroxyapatite (NHA) organic-inorganic hybrid aerogels loaded with stromal cell derived factor-1 (SDF-1) and sclerostin monoclonal antibody (SOST McAb) and investigated their ability to repair steroid-induced osteonecrosis. Rabbit bone marrow mesenchymal stem cells (BMSCs) and human vascular endothelial cells (HUVECs) were used for the *in vitro* study. A rabbit steroid-induced osteonecrosis model was used for the *in vivo* study. The best elastic modulus reached 12.95 ± 4.77 MPa with a mean compressive property of 0.4067 ± 0.084 MPa for the scaffold containing 100% mass fraction. The average pore diameter of the aerogel was 75 ± 18 µm with a porosity of more than 90% (96.4 ± 1.6%). The aerogel-loaded SDF-1 and SOST were released at 40–50% from the material within the initial 3 h and maintained a stable release for more than 21 days. The *in vitro* study showed osteogenesis and vascularization capabilities of the scaffold. The *in vivo* study showed that rabbits received implantation of the scaffold with SOST McAb and SDF-1 showed the best osteogenesis of the osteonecrosis zone in the femoral head. Imaging examination revealed that most of the necrotic area of the femoral head was repaired. These results suggest that this hybrid aerogel scaffold could be used for future steroid-induced osteonecrosis repair.

## Introduction

Steroid-induced osteonecrosis of the femoral head (ONFH) is due to long-term use or a high dose of glucocorticoids in a short time (Wang et al., 2018). Great health-related burdens, including pain and work ability loss, were caused by the disease. Because of the sclerotic zone around the necrotic area of the femoral head, it is difficult for conventional drugs to reach the necrotic area through blood circulation, and which is one of the reasons why there are currently no effective therapeutic drugs for ONFH (García-Gareta et al., 2015; Wang et al., 2016). Through drilling, decompression is the most commonly used surgical method for head protection treatment for osteonecrosis, and two-thirds of patients seek total hip arthroplasty at the end stage (Peng et al., 2018).

An aerogel is a type of special gel that uses gas to replace the liquid in the gel without essentially changing the network structure or volume of the gel itself (Moon et al., 2011). It has the characteristics of a micrometer porous structure, high porosity, and ultralow density. In addition, it has the maximum internal surface area of the same volume in the material at present (Cai et al., 2014; Barrios et al., 2019). Nanocellulose has a wide range of sources, low price, nontoxicity, biodegradability, excellent mechanical properties, and biocompatibility and can serve as an ideal raw material for the synthesis of aerogels (Long et al., 2018). Inorganic-organic hybrid nanocellulose has or exceeds the performance advantages of single-component nanomaterials: 1) Inorganic-organic hybrid nanocellulose aerogel has the advantages of traditional aerogel properties (low density, superior strength). That is, the pore size, strength, and elastic modulus of the material can be adjusted by changing the content of the composition so that it has a wider control range of elastic modulus and excellent mechanical properties, and has the natural advantage of becoming a hard tissue repair material (Kontturi et al., 2018; Wu et al., 2018). 2) Inorganic-organic hybrid nanocellulose aerogels have high biocompatibility and degradability. Cai et al. used natural nanocellulose aerogel microspheres as the medium for coculture with 3T3NIH cells, and the cells were colonized and grew well in the medium in which nanofiber aerogel microspheres were used as the growth skeleton (Cai et al., 2014). 3) The high porosity and specific surface area of inorganic-organic hybrid nanocellulose aerogels can carry more drugs, and the controllable and adjustable drug release rate enables them to be used as carriers of drugs or bone-induced cytokines. The maximum drug-carrying capacity and stable drug release capacity were achieved at a constant volume. Studies have demonstrated that the drug release of aerogel scaffolds can reach a peak quickly and maintain the peak drug dose for a long time (Zhao et al., 2015; Bhandari et al., 2017; Kéri et al., 2020). These superior properties provide a theoretical basis for the application of inorganic-organic hybrid cellulose aerogels in the field of bone defect repair.

In the repair of bone defects, it is necessary to recruit stem cells to gather in the defect area and differentiate in a direction conducive to bone formation. SDF-1 (stromal cell-derived factor-1) is an important chemokine secreted by bone marrow stromal cells that play an important role in progenitor cell homing, hematopoiesis, angiogenesis, and immunity. Studies have shown that SDF-1 binds to CXC chemokine receptor-4 (CXCR4) to activate CXCR4 receptor coupled G protein, to activate the P13K/PKC/NF-kb pathway and other pathways to recruit BMSCs, to promote BMSCs to secrete II collagen and glycosaminoglycan, to induce BMSCs to differentiate into osteoblasts, and to promote new bone formation. SDF-1 can also recruit vascular endothelial progenitor cells (EPCs) to promote angiogenesis and provide support for the repair of bone defects (Takahashi et al., 2020; Tamari et al., 2020). The coding gene of sclerostin encodes a protein polypeptide secreted by osteocytes that inhibits the growth of bone tissue because the expression of SOST is highly tissue specific. Inhibiting the secretion of sclerostin and weakening its activity can increase osteogenesis. At present, it has been confirmed that a specific antibody against SOST can antagonize the effect of SOST, ensure the normal transduction of the classical WNT signaling pathway and the normal physiological function of BMP, and make bone formation activity proceed smoothly (Korn et al., 2019; Scheiber et al., 2019). Nanohydroxyapatite (NHA) and type I collagen are components of bone tissue, and a large number of basic and clinical studies have confirmed the role of nanohydroxyapatite and type I collagen in the process of bone tissue repair. Nanocellulose has a large number of carboxyl groups that can be combined with any organic or inorganic particles through covalent or noncovalent bonds (Dong et al., 2013). Some researchers have successfully prepared new nanocellulose-based hybrid materials with uniform NHA distribution and have shown excellent biocompatibility and biomechanical properties (Jiang et al., 2013; Wang et al., 2019).

In the current study, we aimed to synthesize elastic aerogel scaffolds containing nanocellulose, nanohydroxyapatite, and type I collagen. Meanwhile, we used nanocellulose to carry SDF-1 and SOST McAb to observe the effect of this new material on the proliferation and differentiation of BMSCs and HUVECs *in vitro* and the repair of steroid-induced ONFH *in vivo*.

## Materials and Methods

### Reagents

Information on the reagents used in the current study is listed in Supplementary Table S1.

### Preparation of Scaffolds

**FIGURE 1:**
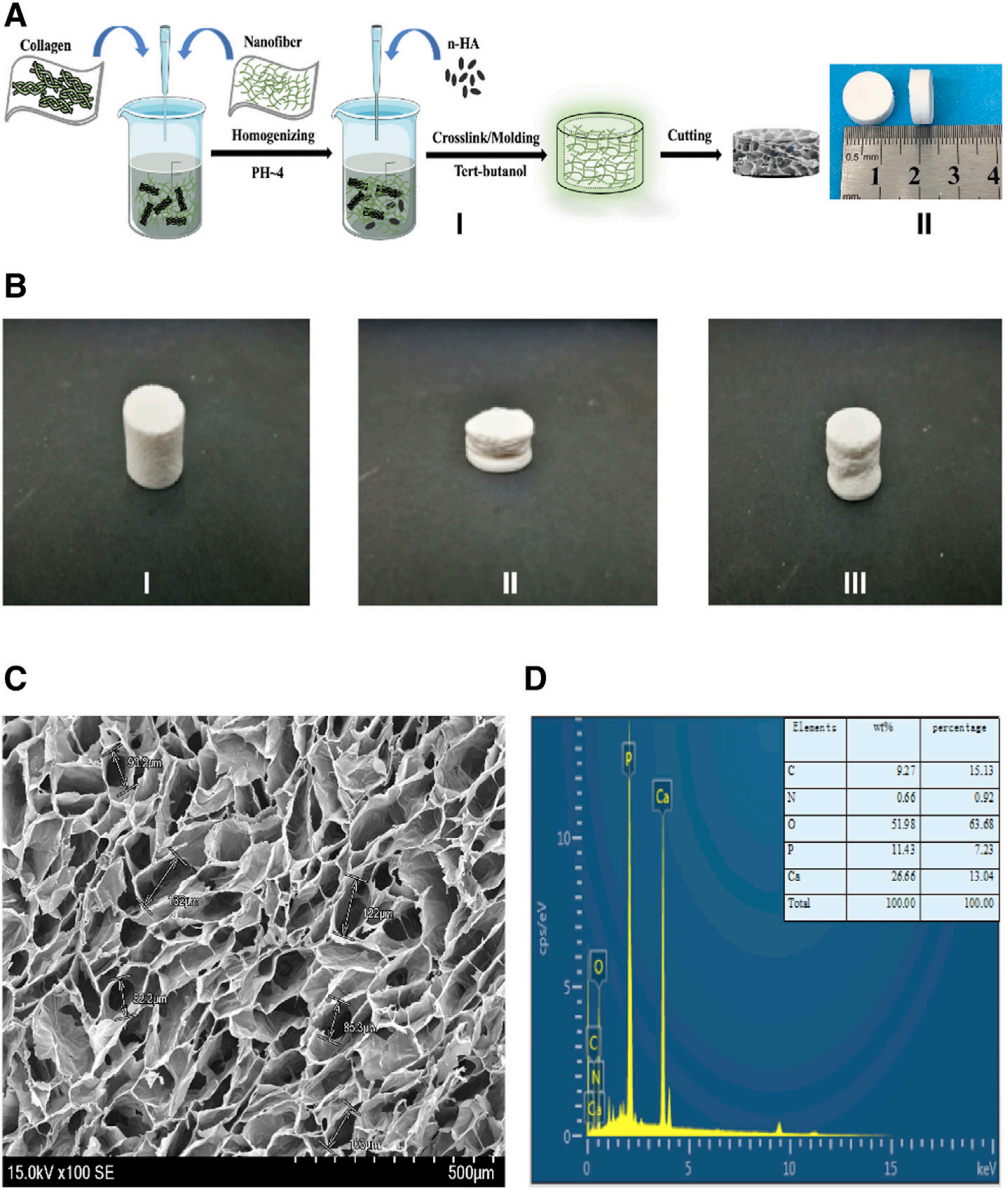
The fabrication process, characterization of the NCF-Col-NHA aerogel scaffold **(A).** The fabrication process and appearance of NCF-Col-NHA aerogel scaffolds. I) show the manufacturing process of aerogel scaffolds: NCF and Col were mixed at a ratio of 2:1, stirred under PH4 conditions, and then added NHA, to get NCF-Col-NHA hydrogel. After replacing the water in the hydrogel with tert-butanol, the NCF-Col-NHA aerogel scaffold was obtained by freeze-drying at-20 °C for 48 h. II) is the finished product of the aerogel scaffold, which is the white porous scaffold **(B).** The shape of NCF-Col-NHA aerogel scaffolds could be restored after compression. **(C)** Morphological observation and elemental analysis of NCF-Col-NHA aerogel scaffolds. Observed under an electron microscope, NCF-Col-NHA aerogel is a porous structure with large and small pores connected **(D).** The elemental analysis of aerogel scaffold, phosphorus (P), calcium (Ca), oxygen (O), and carbon (C) are the main element.

Our previous studies showed that when the ratio of nanocellulose (NCF): collagen (Collagen, Col) = 2: 1, a uniform and stable three-dimensional porous scaffold was formed, the pore structure of the scaffold changed little, and the pore diameter was uniform. NCF and Col at 2:1 were arranged into a 1.0% (w/v) suspension in deionized water, the pH was adjusted to 4, and different contents of NHA (NHA: NCF/Col = 50, 100, 150, and 200%) were added to the NCF/Col mixture and then stirred at 1,000 rpm/min until there was no granular NHA in the mixture. Then, under stirring at 300 rpm/min, γ-(2.3 epoxide) propyltrimethoxysilane was added dropwise to the mixed solution of NCF-Col-NHA, and the cross-linking reaction was continuously stirred for 48 h. The mold rested at 0–44°C for 4 h and then was replaced in tert-butanol solvent for 24 h, during which tert-butanol was replaced every 8 h. Finally, the sample was freeze-dried at −20°C for 48 h to obtain the NCF-Col-NHA aerogel scaffold (Figure 1A).

### Mechanical Properties and Characterization of NCF-Col-NHA Aerogel

The NCF-Col-NHA aerogels were treated with a hairdryer, and a copper sulfate dryer was placed to adjust humidity. The compressive strength of aerogel scaffolds with different NHA contents was tested by a constant loading pressure tester (compression speed 10 N per second, Instron, United States), and NCF-Col-NHA aerogel scaffolds with good mechanical strength were selected to carry SOST McAb + SDF-1. The NCF-Col-NHA aerogel with excellent mechanical strength was packed in separate bags and disinfected with ethylene oxide, and then 40 μl SOST McAb (1 μg/ml), 40 μl SDF-1 (1 μg/ml), 40 μl SOST McAb (1 μg/ml) and 40 μl SDF-1 (1 μg/ml) were dripped under aseptic conditions. After freeze-drying, aerogels loaded with SOST McAb, aerogels loaded with SDF-1, and aerogels loaded with SOST McAb + SDF-1 were obtained. The porosity of the aerogel scaffolds with different NHA contents was measured by the anhydrous ethanol replacement method. The sample was immersed in silica gel in a dryer for 48 h. The specific steps were as follows: the NCF-Col-NHA aerogel was immersed in anhydrous ethanol and weighed 24 h later, and five parallel groups were established to take the average value and calculate the standard deviation. The calculation formula is (W and W_0_ are the initial mass and 24-h mass of the sample, respectively; V and V_0_ are the initial volumes and 24-h volume of anhydrous ethanol, respectively).

The micromorphology and internal structure of the NCF-Col-NHA aerogel were observed by scanning electron microscopy (SEM, Nova SEM450, FEI, United States): longitudinal sections of the NCF-Col-NHA aerogel scaffold were obtained after rapid extraction with liquid nitrogen at low temperature. After drying, the sample was set on the sample table to spray gold. The acceleration voltages of desktop SEM and field emission SEM are 5 and 20 kV, respectively. After SEM scanning, the selected area was scanned by an energy dispersive spectrometer (XSAM800, Kratos), and the surface morphology (pore diameter, porosity) and element composition of the NCF-Col-HA scaffold were analyzed. Aerogel scaffolds with excellent mechanical properties and characterization were selected for follow-up experiments.

### Detection of Degradation and Drug Release Rate of Aerogel Scaffolds in vitro

The NCF-Col-NHA aerogel scaffolds were immersed in a polyethylene plastic pipe containing SBF at a material: liquid ratio of 1:30 (g/ml). The polyethylene plastic pipe was made airtight and placed on a constant temperature shaker at 37°C at a rate of 120 rpm. The degradation experiments were performed *in vitro* for 120 days. The samples were removed and washed with deionized water ultrasound and anhydrous ethanol 3 times at 1, 7, 14, 21, 28, 42, 84, and 110 days. The samples were placed in a vacuum drying oven at 50°C for 12 h and weighed with an electronic balance. The weight of the scaffold is (W_0_) before drying and (W_1_) after drying. The weight loss rate (WL%) was calculated according to the mass loss of the scaffold before and after immersion:

The NCF-Col-NHA aerogel with excellent mechanical strength was packed in separate bags and disinfected with ethylene oxide, and then 40 μl SOST McAb (1 μg/ml), 40 μl SDF-1 (1 μg/ml), 40 μl SOST McAb (1 μg/ml) and 40 μl SDF-1 (1 μg/ml) were dripped under aseptic conditions. After freeze-drying, aerogels loaded with SOST McAb, aerogels loaded with SDF-1, and aerogels loaded with SOST McAb + SDF-1 were obtained. The same volume of materials with different drugs (aerogels loaded with SOST McAb only, aerogels loaded with SDF-1 only, and aerogels loaded with SOST McAb + SDF-1) and the control group (aerogel scaffold without drug-loaded) were placed in a centrifuge tube containing 1 ml PBS and placed in a constant temperature concussion box at 37°C with a vibration frequency of 60 times per minute. At the predetermined time, the leach liquor was taken to detect the concentration of SDF-1 and SOST monoclonal antibodies, and the drug release curve was drawn.

### Biological Properties of Aerogel Scaffolds in vitro

BMSCs from New Zealand rabbits were isolated according to established protocols and cultured in DMEM supplemented with 10% fetal bovine serum and 1% penicillin-streptomycin at 37°C in a 5% CO_2_ incubator. The confluent cells were detached with a 0.25% trypsin mixture, and the isolated cells were subcultured in equal parts. Third-passage cells were employed to conduct the following experiment.

The same amount of aerogel scaffold with different drugs was placed into the DMEM culture, shaken in a constant temperature concussion box at 37°C for 72 h, and then filtered with a 22 μm filter to obtain the extracts. The cytotoxicity test was carried out according to the biological evaluation of GB/T16886.5-2003 medical devices. A BMSC suspension with a density of 1 × 10^4^ cells/ml was added to a 96-well cell culture plate, and different aerogel scaffold extracts were added to the culture medium after cell attachment. Group I was aerogel extract without loading drugs, Group II was the extract of aerogel loaded with SOST McAb, Group III was the extract of aerogel loaded with SDF-1, Group IV extracted from aerogel loaded with SOST McAb + SDF-1, and Group V was the control group, with just the DMEM culture medium. There were ten holes in each group. The cells were cultured at 37°C, 5% CO_2_ saturated humidity constant temperature incubator, and the culture medium was refreshed every 2 days. Quantitative analysis was performed with the CCK-8 kit on the 1st, 3rd, 5th, and 7th days, and the absorbance (OD value) of each well at 450 nm wavelength was detected by an automatic enzyme labeling instrument (Bio–Rad, United States). The relative growth rate (RGR) of BMSCs was estimated according to the OD value to evaluate cytotoxicity. RGR calculation formula: (Ren et al., 2008). According to the methods in the literature, the live/dead assay was carried out in each group after 3 days of culture (Zhang et al., 2019).

BMSCs and HUVCs were seeded on different scaffolds placed in 12-well culture plates at a density of 1 × 10^5^ cells/well, incubated at a constant temperature of 37°C, 5% CO_2_, and saturated humidity in an incubator for 5 h, allowing the cells to adhere to the scaffolds. Group I was the scaffold not loaded with drugs, Group II was the scaffold loaded with SOST McAb, Group III was the scaffold loaded with SDF-1, and Group IV was the scaffold loaded with SOST McAb + SDF-1. Four days later, the scaffolds were placed in 2.5% glutaraldehyde solution, moved to the refrigerator at 4°C for 24 h, and then gently rinsed with PBS solution three times. The material was gradient dehydrated with different concentrations of tert-butyl alcohol solution (50, 70, 90, 95, and 100%) and then vacuum freeze-dried for 24 h. Dried scaffolds were sprayed with gold, and the growth of cells on the surface of aerogel scaffolds was observed by electron microscopy. After being treated according to the standard process, the BMSCs and HUVCs were stained with iFluor555-labeled phalloidin and diaminphenyl indoles (DAPI). The cell morphology was observed by laser confocal microscopy, and the image was analyzed by ImageProPlus software. The cell spreading area and nuclear area of intact cells in the visual field were measured. The ratio of the cell spreading area to the nuclear area was calculated. *Transwell Test*.

The effect of scaffolds on the migration ability of BMSCs and HUVECs was examined by Transwell experiments. Group I was aerogel without drug loading, Group II was aerogel loaded with SOST McAb, Group III was aerogel loaded with SDF-1, Group IV was scaffold loaded with SOST McAb + SDF-1, and Group V was a simple culture medium group. The extract of each group was put into the Transwell chamber. The BMSC or HUVEC suspension (1 × 10^5^ cells/ml, 100 µl) was dripped into the chamber. After 24 h of incubation, the Transwell chamber was removed, and the number of cells migrating through the floor membrane of the small ventricle was calculated. The test was repeated three times.

### HUVEC Tubule Formation Assay

A tubule formation assay was used to determine the ability of extracts of different scaffolds to induce HUVEC tubule formation. The grouping was the same as 2.6. Matrigel was spread in a 15-well plate for tubule formation at 50 µl per well and incubated for 30 min. The HUVEC suspension was prepared with 1.5 × 10^4^ cells per well, and 30 µl of 1% scaffold extract was added. There were 3 wells in each group, and they were incubated for 12 h. Five random visual fields were taken under a light microscope, and the tube volume was analyzed by ImageJ. The upper layer of liquid in the well was carefully absorbed, 50 µl of calcein was added to stain the cells in each well, and the cells were incubated at room temperature for 30 min away from light. Then, a fluorescein isothiocyanate (FITC) filter was used to collect the pictures.

### ALP Activity and Cell Mineralization

P3 rabbit BMSCs were cocultured with different aerogel scaffolds for 7 days (Group I was aerogel without drug loading, Group II was aerogel loaded with SOST McAb, Group III was aerogel loaded with SDF-1, and Group IV was aerogel scaffold loaded with SOST McAb + SDF-1). One milliliter of 0.1% Triton X-100 cell lysate was added to each well and frozen-thawed repeatedly at 37°C and −20°C three times. The obtained cell lysate was transferred into a centrifuge tube and centrifuged at 15,000 r/minute for 5 min. Each group absorbed 30 µl of the supernatant from the centrifuge tube. The ALP measurement was performed according to the operating instructions of the ELISA kit, the OD value was measured at 450 nm by an enzyme labeling instrument, and the OD values in each group were compared.

In the mineralization experiment, we had five groups: Group I was aerogel extract without a drug, Group II was aerogel extract loaded with SOST McAb, Group III was aerogel extract loaded with SDF-1, Group IV was aerogel extract loaded with SOST McAb + SDF-1, and Group V was FBS medium. A third-generation rabbit BMSC suspension with a concentration of 1 × 10^5^ cells/ml was added to the wells (1 ml per well) and cultured at 37°C, 5% CO_2_, and saturated humidity. The culture medium was changed every 2 days, and alizarin red staining was performed after 14 days. Calcium nodules were observed under the microscope.

### In vivo Study

The experimental scheme was approved by the Ethics Committee of West China Hospital of Sichuan University (Ethical approval No. 2020379A, 12.31.2020), and the experimental process strictly complied with the requirements of “Guiding opinions on being kind to Experimental Animals”.

The modified lipopolysaccharide and hormone modeling method was used to establish a model of hormone-induced osteonecrosis in rabbits (Zhao et al., 2020). Firstly, the rabbits were injected with 10 μg/kg of lipopolysaccharide (LPS; Sigma) intravenously. After 24 h, they were injected once a day intramuscularly with 40 mg/kg of methylprednisolone (MPS) for the following 3 days. Penicillin 4,00,000 U was injected intramuscularly into each rabbit before treatment and then continued for 5 days. Four weeks later, the success of the model was assessed by imaging (X-ray, micro-CT) and histological examination. After successful modeling, the rabbits were randomly divided into five groups. After anesthesia, the rabbits were fixed on the operating table. Open the hip joint capsule near the greater trochanter to expose the femoral head, then use a 3.5 mm Kirschner wire to drill a 5 mm depth hole in the junction area of the femoral head and neck toward the necrotic area in the femoral head. The corresponding aerogel scaffold was implanted into the hole by a tweezer according to the experimental design. Aerogel scaffolds without drugs were implanted in Group I, aerogel scaffolds with SOST monoclonal antibody were implanted in Group II, aerogel scaffolds with SDF-1 were implanted in Group III, aerogel scaffolds with SOST monoclonal antibody + SDF-1 were implanted in Group IV, and nothing was implanted in Group V. The implant material was 0.4 cm in diameter with a 0.5 cm high cylinder (Figure 8A). All aerogel scaffolds were disinfected with ethylene oxide at low temperature (Disinfection Center of West China Hospital of Sichuan University). Each rabbit was injected with 4,00,000 U penicillin intramuscularly for three consecutive days. The samples were taken 12 weeks after the operation.

The specimens of the proximal femur were scanned using X-ray and micro-CT (SCANCO Medical AG vivaCT80). The area of the aerogel scaffold was selected as the region of interest (ROI) to form a three-dimensional image. The analysis parameters included bone mineral density (BMD), number of trabeculae (Tb. N), trabecular thickness (Tb. Th.3D), trabecular space (Tb. Sp.3D), and bone volume/tissue volume (BV/TV) (Bodnyk et al., 2020). The NCF-Col-NHA aerogel region in the scanned layer is reconstructed according to the scaffold, the part of bone growth in the material, and the density of the cavity with different grayscale and marked with different colors.

The removed femoral head was fixed with 10% paraformaldehyde solution and then cut and polished with a slicer to obtain thin slices with a thickness of approximately 20–30 μm, which were stained with HE and methylene blue-magenta. After vacuum critical drying, the tissue sections of each group were sprayed with gold. The bonding degree between the implanted aerogel scaffold and the host bone was observed bySEM.

### Osteogenic and Angiogenic Gene Expression

Quantitative real-time polymerase chain reaction (qRT-PCR) was conducted to determine osteogenic and angiogenic gene expression. Total RNA was extracted from the femoral head using TRIzol reagent and then reverse-transcribed according to the synthesized *VEGF*, *ALP*, and *BMP-2* gene primers by a reverse transcription kit (Supplementary Table S2). Then, qRT-PCR detection was carried out. Glyceraldehyde 3-phosphate dehydrogenase (*GAPDH*) was used as an internal reference for data standardization. Compared with the normal femoral head, the relative expression level of target gene mRNA was analyzed and calculated, and the expression of VEGF, ALP, and BMP-2 protein was detected by Western blot according to the standard flow.

### Health Status of Organs

12th weeks after the operation, the liver, spleen, and kidney tissues of each group were taken and stained with HE, and the health status of each organ was observed.

### Statistical Analysis

All of the experiments were conducted in triplicate. One-way ANOVA followed by the least significant difference (LSD) test and Student–Newman–Keuls (SNK) test were performed to determine significant differences using IBM SPSS 20.0. *p* < 0.05 was considered statistically significant.

## Results

### Mechanical Properties and Characterization of the NCF-Col-NHA Aerogel Scaffold

Aerogels with different mass fractions of NHAs have different compressive properties (50% HA: 0.3550 ± 0.0353 MPa; 100% HA: 0.4067 ± 0.084 MPa; 150% HA: 0.3641 ± 0.0506 MPa; 200% HA: 0.4262 ± 0.0209 MPa) and elastic modulus (50% HA: 4.25 ± 0.41 MPa; 100% HA: 12.95 ± 4.77 MPa; 150% HA: 8.16 ± 2.80 MPa; 200% HA: 9.36 ± 2.48 MPa). The composite aerogel scaffold with 100% NHA content recovered 95% of its deformation after compression (Figure 1B). Under the electron microscope, the NCF-Col-NHA composite scaffold materials with different HA contents are all through-pore structures with large pores and small pores coexisting. With increasing HA mass ratio, the pore size of the NCF-Col-NHA composite materials gradually decreased (50% HA: 90 ± 15 μm; 100% HA: 75 ± 18 μm; 150% HA: 60 ± 11 μm; 200% HA: 40 ± 13 µm). When the mass ratio of NHA was 50 and 100%, the material maintained a relatively good porous structure, but with the increase in the HA mass ratio, the pore structure of the material gradually deformed or even collapsed, and the porosity of the NCF-Col-NHA aerogel with 150 and 200% HA contents showed an obvious downward trend (50% HA: 97.3 ± 1.5%; 100% HA: 96.4 ± 1.6%; 150% HA: 89 ± 3.2%; 200% HA: 80 ± 2.3%). The composite scaffold with a 100% NHA ratio has the best mechanical strength and good porous structure, so we chose it for follow-up experiments. EDS elemental analysis showed that the main components of aerogel scaffolds included phosphorus (P), calcium (Ca), oxygen (O), and carbon (C) (Figures 1C,D).

### Degradation of Aerogel Scaffolds in vitro

**FIGURE 2:**
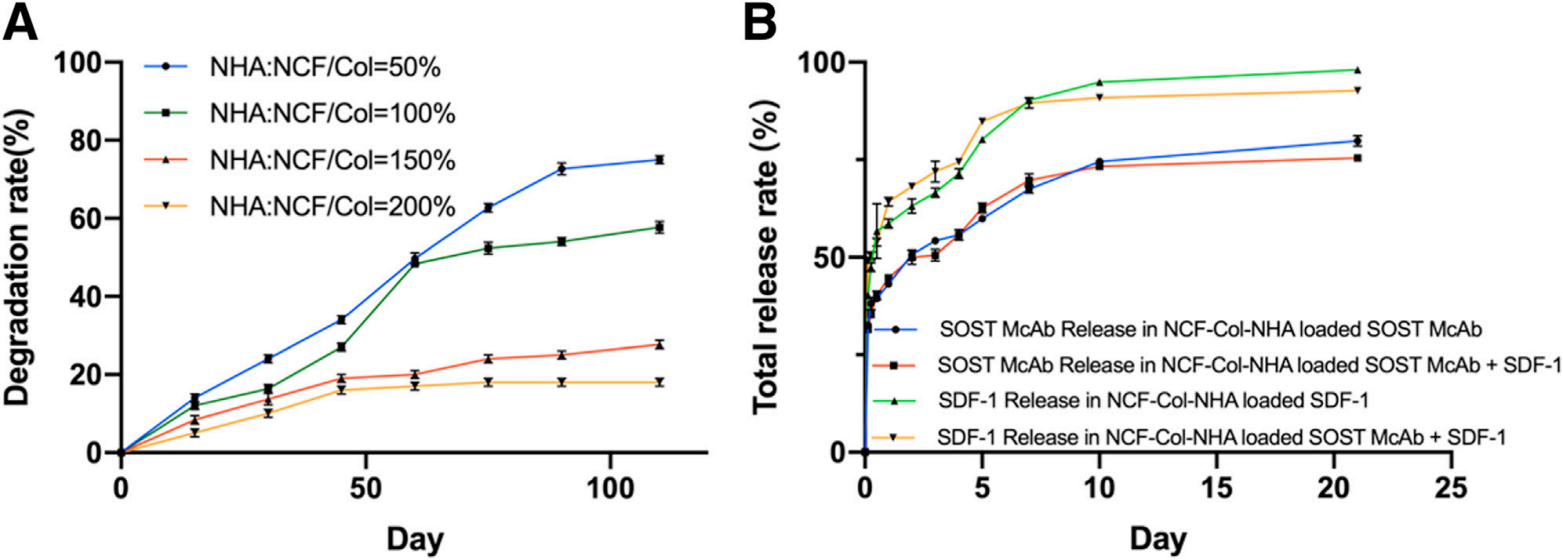
The degradation curve and drug release curve of the NCF-Col-NHA aerogel scaffolds *in vitro*. **(A)** *In vitro* degradation curve of NCF-Col-NHA aerogel scaffolds with different NHA content. The less NHA content, the faster the material degrades **(B).** Drug release curve of aerogel scaffolds loaded with different drugs. There was no difference in release rate between SOST McAb and SDF-1, whether co-loaded or individually loaded on aerogel scaffolds.

The degradation rate of scaffolds decreased with increasing NHA content. The early stage of scaffold degradation is mainly swelling and cellulose disintegration. When the proportion of NHA is 50%, it is degraded to a certain extent, and cracks appear on the surface and interior of the material so that a large number of NCFs come into contact with simulated body fluids and swell rapidly, but the disintegration value is not reached within the range of degradation research. When the proportion of NHA was 100%, the degradation rate decreased slowly with increasing time, the NCF swelling and the degradation of Col and NHA were almost synchronized, and the degradation rate was relatively slow. For the NCF-Col-NHA aerogel with 150 and 200% NHA content, because of a large number of Ca^2+^ ions in NHA, NHA reacted with -OH in NCF to form Ca-O bonds or O-Ca-O bonds, which made the tight combination of NCF and HA difficult to disintegrate. At the same time, NHA reduced the hydrophilicity of NCF, prevented the water molecules in SBF from entering the interior, and reduced the degradation rate and degradation amount. The aerogel scaffold with 100% NHA had the most suitable degradation rate (Figure 2A).

### Drug Release Rate *in vitro*

There was no significant difference in the appearance of aerogel scaffolds loaded with different drugs, and all of them were milky white porous scaffolds. The drug release curve of the loaded NCF-Col-NHA aerogel *in vitro* shows that 40–50% of the SDF-1 and SOST McAbs were released from the material within 3 h and maintained a stable release rate in the following 21 days, and 70–80% of the drugs were involved in drug release on the 21st day. Drug release could be maintained for a longer time after Day 21. There was no difference in the release rate of SOST McAb and SDF-1 coloaded or separately loaded with the scaffolds (Figure 2B).

### *In vitro* Biological Performance

**FIGURE 3:**
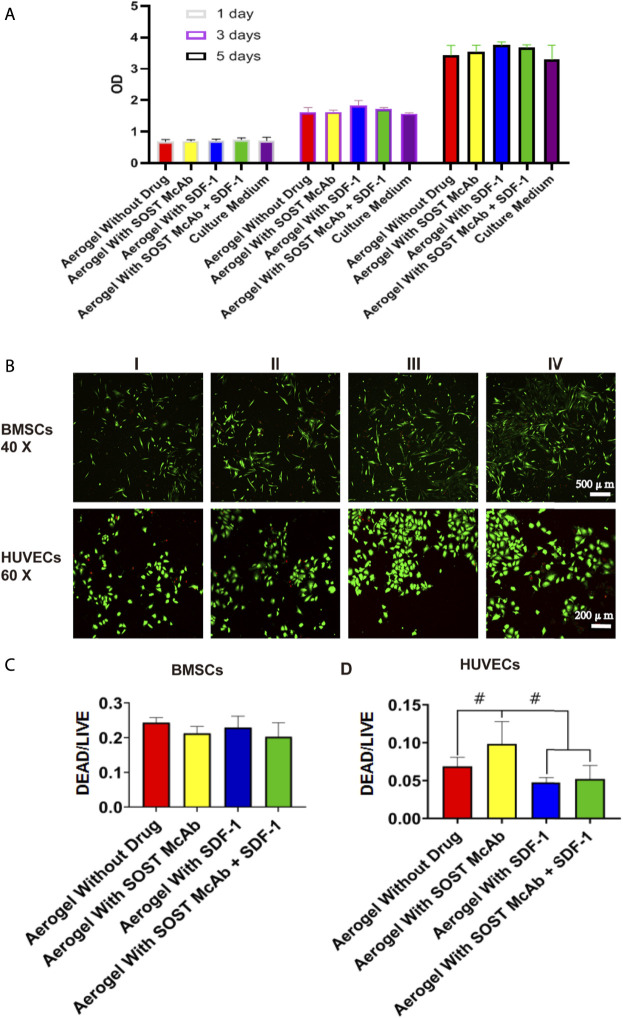
The cell activity evaluation and the result of live/dead assay of the NCF-Col-NHA aerogel scaffold in vitro. Cell activity evaluation. When the extract of NCF-Col-NHA aerogel scaffolds with different drugs was co-cultured with BMSCs, the relative proliferation rate of BMSCs was between 97% and 113% **(A)**, these aerogel scaffolds had no cytotoxicity **(B–D)**. The result of live/dead assay. The BMSCs and HUVECs grow well on the aerogel scaffolds, the ratio of dead/living cells is lower than 10%. Group I, aerogel scaffold without loading drugs; Group II, aerogel scaffold loaded with SOST McAb; Group III, aerogel scaffold loaded with SDF-1; Group IV, aerogel scaffold loaded with SOST McAb + SDF-1. BMSCs, bone marrow mesenchymal stem cells; HUVECs, Human umbilical vein endothelial cells. #, *p* < 0.05.

After coculture of BMSCs and the extract of different aerogel scaffolds, the RGR measured by the CCK-8 method was between 97 and 113%, and the scaffolds of each group met the toxicity requirements of biological implant materials. After BMSCs and HUVECs were grown on different groups of NCF-Col-NHA aerogels for 4 days, the living and dead cells on the materials were observed by live/dead assay. The dead cells were stained red, and the live cells were stained green. Both BMSCs and HUVECs grew well on the NCF-Col-NHA aerogel, and the ratio of dead cells was less than 10%. The ratio of HUVEC-dead cells in the aerogel group loaded with SOST monoclonal antibody was 9.94 ± 3%, which was slightly higher than that in the other groups (*p* < 0.05). There was no significant difference in the ratio of live/dead cells among the groups (*p* > 0.05) (Figure 3).

**FIGURE 4:**
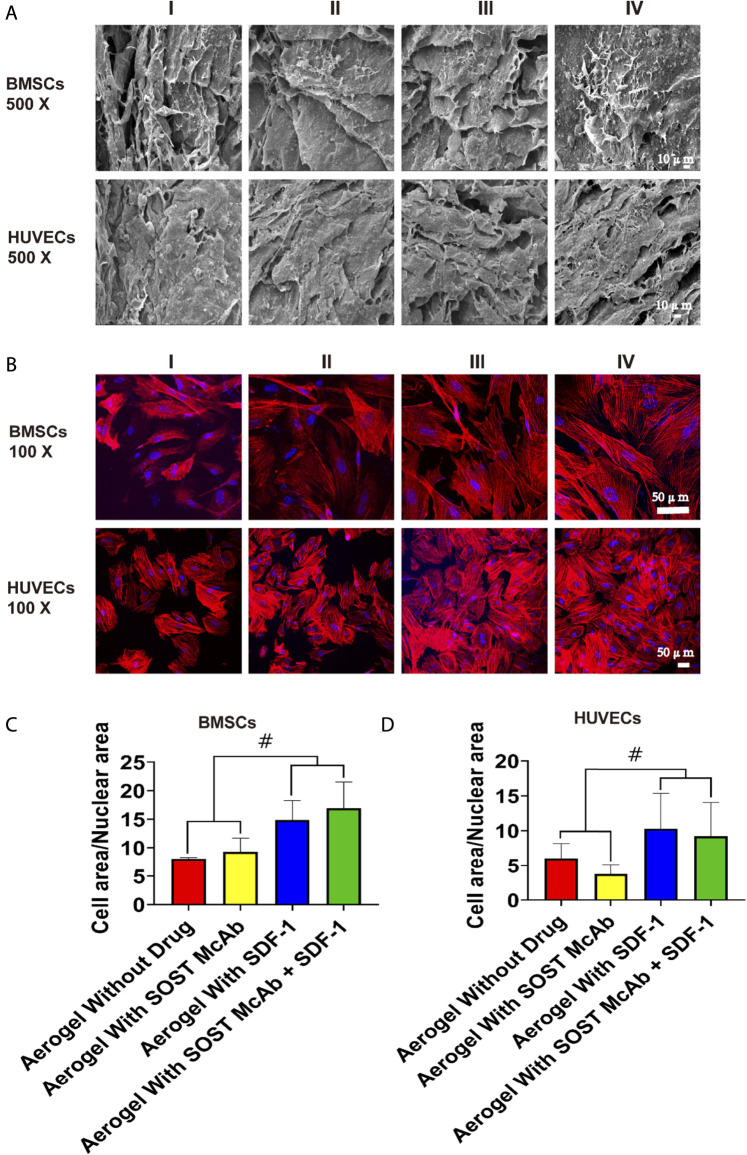
The biocompatibility of the NCF-Col-NHA aerogel scaffold with BMSCs and HUVECs *in vitro*. **(A)** Under the electron microscope, BMSCs and HUVECs can adhere to the surface of aerogel scaffolds loaded with different drugs **(B–D)**. Observation on the morphology of cells on the surface of NCF-Col-NHA aerogel scaffolds. The results of iFluor555 labeled phalloidine and diaminphenyl indoles stain and cell area/nuclear area calculation showed that SDF-1 was beneficial to the adhesion and growth of BMSCs and HUVECs on the surface of the scaffolds. Group I, aerogel scaffold without loading drugs; Group II, aerogel scaffold loaded with SOST McAb; Group III, aerogel scaffold loaded with SDF-1; Group IV, aerogel scaffold loaded with SOST McAb + SDF-1. BMSCs, bone marrow mesenchymal stem cells; HUVECs, Human umbilical vein endothelial cells. #, *p*<0.05.

After BMSCs and HUVECs were cultured on different NCF-Col-NHA aerogels for 4 days, SEM revealed that both BMSCs and HUVECs could adhere to the aerogel scaffolds and grow pseudopodia. Compared with HUVECs, BMSCs had more and longer pseudopodia. The aggregation of cells on drug-loaded aerogel scaffolds was more obvious. There were more cells attached to SDF-1 aerogels and SOST McAb + SDF-1 aerogels, which may be related to the effect of SDF-1 on cell homing (Figure 4A). The area ratio of cytoplasm to nucleus was analyzed by Image software. The area ratio of cytoplasm to the nucleus in the aerogel group loaded with SDF-1 and SOST McAb + SDF-1 was 14.88 ± 3.39 and 16.95 ± 4.58, respectively, which was higher than that without drug loading (8.07 ± 0.20) and that loaded with SOST McAb (9.30 ± 2.42). The cytoplasmic/nuclear area ratios of HUVECs loaded with SDF-1 and SOST McAb + SDF-1 aerogels were 10.30 ± 5.07 and 9.22 ± 4.83, respectively, which were higher than those of HUVECs loaded with unloaded drugs (6.02 ± 2.11) and SOST McAb (3.76 ± 1.38) (Figures 4B–D).

Four hours after inoculation, the cells passing through the Transwell chamber could be seen in all groups. The average number of cells passing through the small ependyma in the group without drug aerogel was 122 ± 16 and 85 ± 9, respectively, which was lower than that in the group with drugs (*p* < 0.05). There was no significant difference between aerogel scaffolds loaded with different drugs.

**FIGURE 5:**
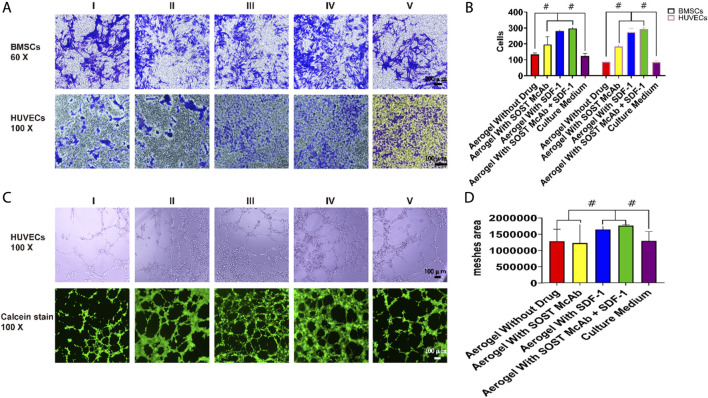
The results of Transwell test and tubule formation assay. **(A,B)** Results of cell staining and cell count in Transwell test. The Transwell test shows that the extracts of aerogel scaffolds containing drugs were better than that of aerogel without drugs and common culture medium. Each bar represents a group, better Transwell test results were obtained in the extract of drug-loaded aerogel scaffolds **(C,D).** The result of HUVECs tubule formation Assay. Calcein staining results showed that the aerogel scaffold with SDF-1 had the best effect of forming tubules, and the histogram of analysis of meshes area showed the same results, the difference between groups was statistically significant. Group I, aerogel scaffold without loading drugs; Group II, aerogel scaffold loaded with SOST McAb; Group III, aerogel scaffold loaded with SDF-1; Group IV, aerogel scaffold loaded with SOST McAb + SDF-1; Group V, the control group with simple culture medium. BMSCs, bone marrow mesenchymal stem cells; HUVECs, Human umbilical vein endothelial cells. #, *p*<0.05.

Six hours after inoculation, HUVECs were arranged into a network structure. The areas of aerogels loaded with SDF-1 and SOST McAb + SDF-1 were 1650287.00 ± 99375.23 and 1768436.67 ± 29859.84, respectively, which were higher than those of the SOST McAb group (1231132.75 ± 570126.52) and unloaded drug group (1289030.50 ± 369031.77) (Figure 5).

### Osteogenic Differentiation

**FIGURE 6:**
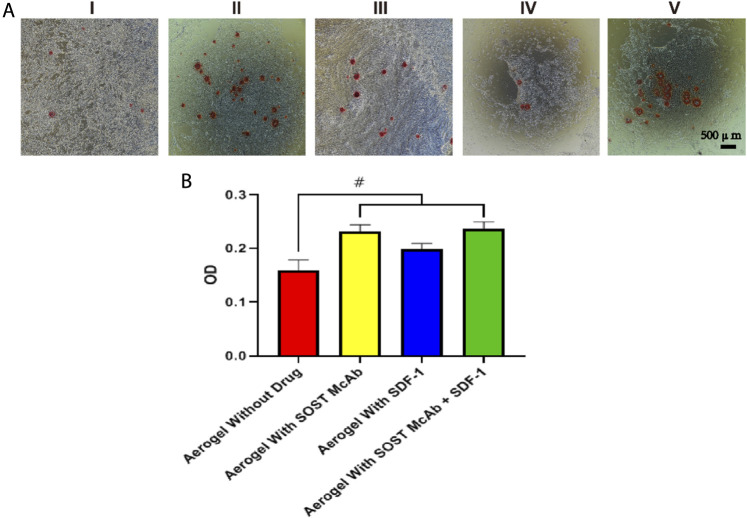
The result of ALP activity and cell mineralization. **(A)** The results of alizarin red staining **(B)** alkaline phosphatase detection. The results showed that the aerogel scaffold with SOST Macb had a better osteogenic differentiation effect on BMSCs. Group I, aerogel extract without loading drugs; Group II, the extract of aerogel loaded with SOST McAb, Group III, the extract of aerogel loaded with SDF-1; Group IV, extracted from aerogel loaded with SOST McAb + SDF-1; Group V the control group with simple culture medium. #, *p*<0.05.

ELISA kits were used to detect the ALP content of cell lysates after coculture of aerogel scaffolds and BMSCs in each group. The OD value measured by the enzyme labeling instrument showed that the unloaded drug aerogel group was 0.16 ± 0.02, which was lower than those aerogel scaffolds with drugs (*p* < 0.05). The OD values of the McAb and McAb + SDF-1 aerogel groups were 0.23 ± 0.01 and 0.247 ± 0.02, respectively, which were higher than those of the SDF-1 aerogel scaffold (*p* < 0.05). The results of alizarin red staining showed that there were more calcium nodules in the SOST McAb and SOST McAb + SDF-1 groups (Figure 6).

### *In vivo* Experiments

**FIGURE 7:**
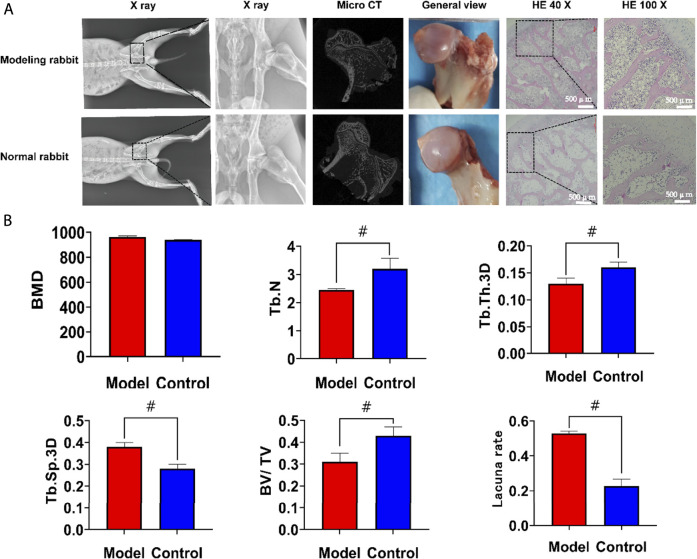
The results of modeling of the steroid-induced osteonecrosis model in New Zealand rabbits. **(A)** After modeling, there was no collapse of the femoral head. Compared with the normal rabbits, the X-ray, Micro CT and HE staining showed that the trabeculae in the femoral head became thinner and the empty lacunae increased **(B).** Statistical results of regions of interest. The number of trabeculae and the bone volume/tissue volume was significantly lower than that in the normal group, the trabecular space was higher than the normal group but there was no significant difference in the mineral density between the two groups. The empty bone lacuna rate in the model group was more than 50%, which was significantly higher than that in the control group. HE, hematoxylin-eosin staining; BMD, bone mineral density; Tb. N, number of trabeculae; Tb. Th.3D, trabecular thickness; Tb. Sp.3D, trabecular space; BV/TV, bone volume/tissue volume. VEGF, vascular endothelial growth factor; BMP2, Bone Morphogenetic Protein 2; ALP, alkaline phosphatase. #, *p*<0.05.

To further study the biological performance of the aerogel scaffolds, we conducted an *in vivo* animal test for 12 weeks to confirm the bone regeneration ability of the scaffolds. In this study, 80 New Zealand rabbits were injected intramuscularly to induce steroid-induced ONFH. On the 2^nd^ day after drug injection, most rabbits showed lethargy, reduced food intake, hair removal, and reduced activity, and some rabbits began to develop diarrhea. One week later, the mental state of the rabbit began to recover, and the intake of food gradually increased, but the activity of the rabbit was still less than that before the drug injection. Within 4 weeks, 20 rabbits died of upper gastrointestinal ulcers, perforation, diarrhea, and so on. When taking the model rabbit specimens, osteoporosis was found around the femoral head, the femoral cortex became thinner, and the color of the femoral head was darker than that of the normal rabbit, but there was no collapse of the femoral head. The X-ray results showed that the density of the femoral head in the model group was uneven, the trabeculae were blurred, and the trabeculae were partially interrupted. Micro-CT showed that the number of trabeculae in the model rabbits decreased, the width between the trabeculae increased, the cortex of the femoral neck became thinner, and the femoral head and trochanter were osteoporotic. The number of trabeculae (2.45 ± 0.04 /mm^3^) and the bone volume/tissue volume (0.31 ± 0.04) were lower than those in the normal control group (*p* < 0.05). The trabecular space (0.38 ± 0.02 mm) was higher than that of the control group (0.28 ± 0.02) (*p* < 0.05), and there was no significant difference in the mineral density between the two groups. HE staining showed that the cartilage matrix and cells in the model group were normal, and the number of adipocytes and inflammatory cells in the bone trabeculae increased. In addition, the number and density of empty bone lacunae increased. The empty bone lacuna rate in the model group was 52.88 ± 1.19%, higher than that in the control group (22.73 ± 3.87%), *p* < 0.05. Steroid-induced osteonecrosis was successfully created in New Zealand rabbits to establish the animal model (Figure 7).

**FIGURE 8:**
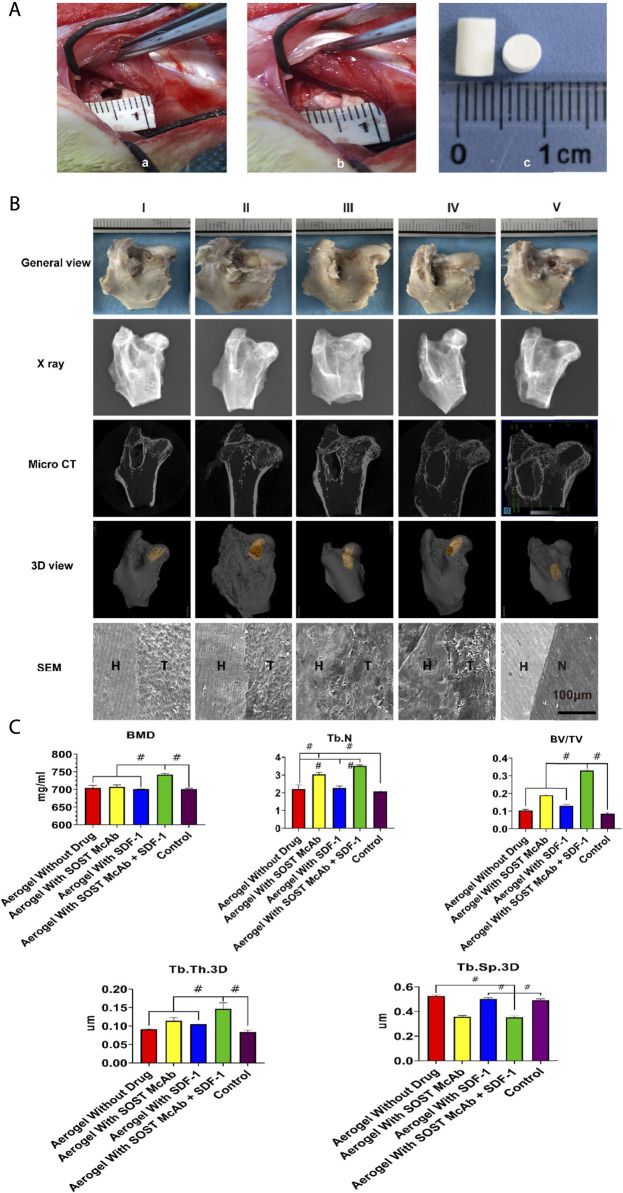
The process and the results *in vivo* experiments. **(A)** the process *in vivo* experimental operation. The aerogel scaffold was implanted after drilling holes at the junction of the head and neck of the femur in rabbits. a) shows the 4 mm diameter of the hole; b) shows the implantation of the aerogel scaffold at the drill hole; c) is the diameter of the implanted aerogel stent, which is 5 mm **(B)**. Observation of New Zealand rabbits implanted with different aerogel scaffolds. There was no obvious collapse of the femoral head in all groups. X-ray and Micro CT showed that there were different degrees of new bone formation in the osteonecrosis area in the aerogel scaffold implanted group (I-IV), while in the group only drilled without aerogel implantation (V), only a small amount of new bone formed at the beginning of the hole, and the osteonecrosis area was not repaired. Under the electron microscope, there were different degrees of integration between aerogel scaffolds and host bone in the I-IV group, among which the group III and IV were the most obvious. There was no new bone formation in the osteonecrosis area of the group without aerogel scaffolds **(C)**. Results of Micro CT region of interest analysis. The bar graph shows that the group of aerogel scaffolds with SOST McAb + SDF-1 has the best bone quality. Group I aerogel scaffold without loading drugs, Group II aerogel scaffold loaded with SOST McAb, Group III aerogel scaffold loaded with SDF-1, Group IV aerogel scaffold loaded with SOST McAb + SDF-1, Group V the control group without scaffold implant, Group VI rabbit without surgery. H marked as the host bone, T marked as the aerogel scaffold, and N marked as the tunnel with granulation tissue. BMD, bone mineral density; Tb. N, number of trabeculae; Tb. Th.3D, trabecular thickness; Tb. Sp.3D, trabecular space; BV/TV, bone volume/tissue volume. #, *p*<0.05.

12th weeks after the operation, there was no collapse of the femoral head in any of the groups. There were still bone defects of the femoral head in the group without an aerogel scaffold implant, and the bone defects were repaired in the other four groups. X-ray and micro-CT showed that the bone defect was not completely repaired in the femoral head of the group without implanted materials. There were different degrees of high-density osteogenic images in the drilling area of the femoral heads in the other four groups. The area of aerogel implanted into the femoral head was selected as the ROI, and the analysis results showed that in the SOST McAb + SDF-1 aerogel group, the BMD was 742.14 ± 3.83 mg/ml, the trabecular thickness was 0.14 ± 0.02 mm, and the BV/TV was 0.33 ± 0.01, which was higher than those in other groups (*p* < 0.05), while the trabecular space (0.35 ± 0.01 mm) was lower than that in the other groups (*p* < 0.05). The number of bone trabeculae in the SOST McAb aerogel group and the SOST McAb + SDF-1 aerogel group was 3.04 ± 0.10/mm^3^ and 3.51 ± 0.06/mm^3^, respectively, which was higher than that in the other three groups (*p* < 0.05) (Figures 8B,C).

**FIGURE 9:**
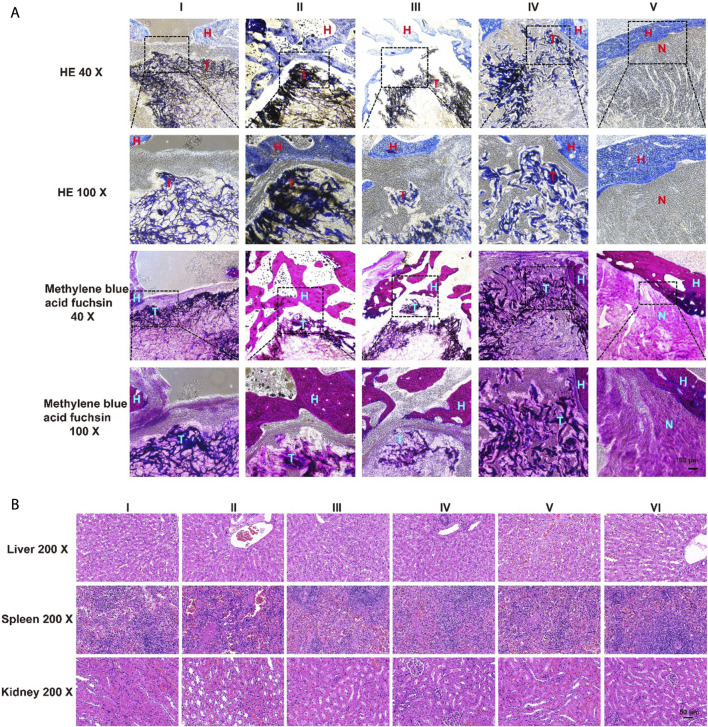
The results of HE and methylene blue acid fuchsin staining and safety monitoring. **(A)** There were obvious bone formation and new bone formation at the junction between the aerogel scaffold and host bone. The ability of the aerogel scaffold carrying SOST McAb + SDF-1 to promote new bone formation was significantly higher than that of other groups **(B)**. Results of HE staining of visceral organs. 12 weeks after the operation, no pathological abnormality was found in the liver, spleen, and kidney of the rabbits in each experimental group (I-IV) compared with the group without scaffold unimplanted (V) and the of the unoperated group (VI). Group I aerogel scaffold without loading drugs, Group II aerogel scaffold loaded with SOST McAb, Group III aerogel scaffold loaded with SDF-1, Group IV aerogel scaffold loaded with SOST McAb + SDF-1, Group V the control group without scaffold implant, Group VI rabbit without surgery. H marked as the host bone, T marked as the aerogel scaffold, N marked as the tunnel with granulation tissue. HE, hematoxylin-eosin staining.

**FIGURE 10:**
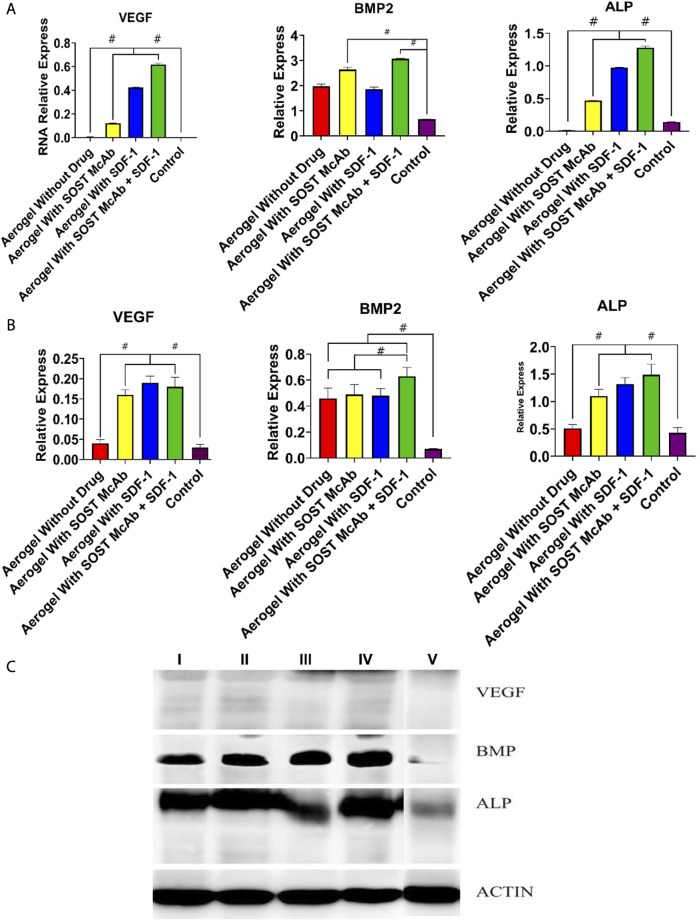
Results of PCR and Western blot detection. **(A)** The results of PCR of VEGF, BMP2, and ALP **(B,C)**. The results of Western blot of VEGF, BMP2, and ALP. The bar graph showed that the aerogel loaded with SOST McAb and SDF-1 had the best effect on promoting new bone and neovascularization. Group I aerogel scaffold without loading drugs, Group II aerogel scaffold loaded with SOST McAb, Group III aerogel scaffold loaded with SDF-1, Group IV aerogel scaffold loaded with SOST McAb + SDF-1, Group V the control group without scaffold implant. VEGF, vascular endothelial growth factor; BMP2, Bone Morphogenetic Protein 2; ALP, alkaline phosphatase. #, *p*<0.05.

The results of HE staining showed that there was obvious new bone formation at the junction between the aerogel scaffold and host bone. In the group with the SOST McAb + SDF-1 aerogel scaffold, the bone grew deeper, and new bone could be observed in the center of the aerogel scaffold. However, in the group without aerogel scaffold implantation, there was only granulation tissue filling in the drilled tunnel but no discernible new bone formation. After methylene blue acid fuchsin staining, the aerogel containing hydroxyapatite was stained purplish red, the new bone tissue was light red, and the host bone tissue was red. The group implanted with the SOST McAb + SDF-1 aerogel scaffold had much more new bone formation, and the ability to encourage bone formation and repair bone defects was significantly stronger than that of the other groups (Figure 9A). SEM observation showed that the growth of new bone could be seen at the junction between aerogel scaffolds and host bone, and the aerogel scaffold with SDF-1 and aerogel scaffold with SOST McAb + SDF-1 was the most obvious (Figure 8B). The PCR results showed that the VEGF gene expression in the simple drilling decompression group and unloaded drug aerogel group was 0.002 ± 0.001 and 0.005 ± 0.001, respectively, which was lower than that in the other three groups (*p* < 0.05). The expression of the ALP gene in the simple drilling decompression group and the unloaded drug aerogel group was 0.143 ± 0.001 and 0.015 ± 0.001, respectively, which was also lower than that in the other three groups (*p* < 0.05). The expression of BMP-2 in the SOST McAb-loaded group and SOST McAb + SDF-1 aerogel group was 2.63 ± 0.09 and 3.07 ± 0.02, respectively, which was higher than that in the other groups (*p* < 0.05). Analogous results were obtained by Western blot detection. The aerogel scaffold loaded with SOST McAb + SDF-1 had the best effect on promoting new bone and neovascularization (Figure 10).

At 12 weeks after the operation, there were no pathological abnormalities in the liver, spleen, or kidney of rabbits with aerogel scaffolds compared with rabbits without aerogel scaffolds and rabbits without surgery. It was confirmed again that the NCF-Col-NHA aerogel scaffold loaded with SOST McAb and SDF-1 had good biosafety (Figure 9B).

## Discussion

The main purpose of this study was to design and make a porous composite scaffold with elasticity, basic recovery of deformation after compression, and the ability to induce osteogenesis and angiogenesis. This aerogel scaffold composed of nanohydroxyapatite, collagen, and nanocellulose was fabricated by improving the aerogel manufacturing process. Compared with traditional bone substitute materials, this new aerogel scaffold has the characteristics of traditional bone substitute materials; that is, it has a three-dimensional porous structure, bone conduction, and bone induction, so it is suitable for bone growth. The ideal scaffold for bone regeneration should obtain good mechanical properties. The compressive strength of the new composite scaffold developed in this study is 12.95 ± 4.77 MPa, which is within the range of compressive strength of cancellous bone (2–220 MPa) (Bose et al., 2012) and is suitable for filling cancellous bone defects of the femoral head. In our previous study, it was found that the higher the content of NHA was, the stronger the mechanical properties of the scaffold, but it affected the anti-deformation ability and porosity of the material. When the ratio of NHA to NCF-Col was 1:1, the scaffold had better mechanical strength and pore structure. The related results will be emphasized in another paper of our team. Previous studies have considered that the pore size of the most suitable material for bone growth is 100–4,400 μm, but some recent research results challenge these data. They think that micropores with pores lower than 100 µm or even nm are also suitable for bone growth, and smaller pores even show a better ability to induce bone growth (Eggli et al., 1988; Itälä et al., 2001; Bose et al., 2012). The average pore size of this new material is 75 ± 18 µm. The *in vitro* and *in vivo* results confirmed that pore sizes lower than 100 µm are suitable for bone growth. In addition, this innovative material has two outstanding characteristics: one is that it can quickly restore deformation after compression and has good plasticity, and the shape of the material can be restored to approximately 95% after compression. The scaffold can quickly restore deformation after being compressed and implanted into the bone defect area through a small orifice, and the volume of the material will be expanded to a certain extent after absorbing water or blood, fully filling the bone defect area. Second, nanocellulose acetate has a large number of carboxyl groups and a large internal surface area, which can carry more osteogenic and angiogenic drugs at the same volume (Ulker and Erkey, 2014). Our scaffolds can bind SDF-1 and SOST antibodies well and can be confirmed to have long-term and stable release, which is helpful to promote the repair of bone defects.

Excellent bone regeneration materials should have good histocompatibility. The nanocellulose acetate, nanohydroxyapatite, and collagen we selected are all materials with appropriate safety, and hydroxyapatite and type I collagen are the normal components of bone tissue (Liu et al., 2016). *In vitro*, it was found that BMSCs and HUVECs could adhere, aggregate, and exert their respective biological activities on the surface of the materials. Depending on the CCK-8 results, the proliferation of BMSCs increased on the composite aerogel scaffolds loaded with SOST monoclonal antibody and SDF-1. BMSCs and HUVECs were cocultured with scaffolds for 4 days, and the staining results of live/dead cells also showed that the scaffolds had good biocompatibility. Aerogel scaffolds loaded with SOST monoclonal antibody and SDF-1 are more conducive to cell adhesion, which provides a helpful beginning for local osteogenesis, angiogenesis, and bone defect repair centered on scaffold materials. In addition, the results of the Transwell experiment and tubule formation experiment also confirmed that the aerogel scaffold loaded with SOST monoclonal antibody + SDF-1 was more conducive to cell growth and biological activity expression than the control scaffold. This is of great importance to the repair of bone defects. *In vitro*, whether cocultured with SOST McAb + SDF-1 composite aerogel scaffold or adding composite scaffold extract to the culture medium, BMSCs showed better osteogenic activity than other groups. In this study, to more intuitively observe the effect of aerogel scaffolds with drugs on the osteogenic differentiation of BMSCs, we did not choose an osteogenic culture medium but used a common culture medium. After 14 days of culture, alizarin red staining of the five groups showed that the aerogel scaffolds carrying SOST monoclonal antibody and SOST monoclonal antibody + SDF-1 had a better effect on promoting the differentiation of BMSCs into osteoblasts than the other three groups.

One of the problems in repairing large bone defects is that it is difficult to form blood vessels in the central area of bone defects. Through the bone conduction of scaffold materials, the new bone tissue can repair and reconstruct the bone defect area while the scaffold is degraded gradually. However, due to insufficient blood supply in the central area of the bone defect, it is unable to provide enough nutrition for the new bone and eventually leads to the failure of bone defect repair (Wendt et al., 2006; Johnson et al., 2011). SDF-1 can recruit stem cells such as bone marrow stromal cells and vascular progenitor cells through the homing effect, and SOST can increase osteogenesis through the osteogenic effect (Suen et al., 2015; Janssens et al., 2018). The results showed that the aerogels loaded with SDF-1 had an obvious effect on the migration of HUVECs and BMSCs, and the aerogels loaded with SOST had an observable osteogenic effect. The aerogel scaffold loaded with SOST monoclonal antibody and SDF-1 can cause stem cells with osteogenic and angiogenic abilities to gather at the aerogel scaffold and differentiate in a direction conducive to osteogenesis and angiogenesis. Neovascularization provides sufficient nutritional support for the central area of the bone defect and timely transport of metabolic waste, which is beneficial to the repair of bone defects, providing sufficient evidence for *in vivo* research.

There are a few difficulties in establishing a model of steroid-induced osteonecrosis in New Zealand rabbits. The combined use of lipopolysaccharide and high-dose glucocorticoids caused diarrhea, digestive tract ulcers, and death in some experimental animals (Powell et al., 2011; Xi et al., 2017). In our study, we reduced the death of experimental animals by giving proton pump inhibitors to the rabbits. After the establishment of the model, we confirmed the success of the model of ONFH by imaging and histological examination. The successful femoral head was drilled, and the scaffold material was implanted. This new composite scaffold perfectly realizes the assumption of implanting large-diameter filling materials through small diameter holes in the experiment. We utilized a 4 mm electric drill to obtain a decompressed bone tract and implanted composite scaffolds with a diameter of 5 mm to achieve good filling of bone defects. 12th weeks after the operation, no collapse of the femoral head was discovered in the rabbits by specimen observation and X-ray examination, indicating that the drilling operation controlled the disease progression of ONFH to a certain extent. The MicroCT results showed that there were more new bones in the femoral head of the experimental animals with aerogel scaffolds than in those with simple drilling. The results of RIO analysis showed that the bone repair effect of the composite aerogel scaffolds with SOST monoclonal antibody and SDF-1 was the best. Histological staining (HE staining and methylene fuchsin staining) confirmed that there was more new bone formation in the bone defect area when implanted with the SOST monoclonal antibody + SDF-1 composite aerogel scaffold. SEM showed that the fresh bone grew staggered at the interface between the aerogel scaffold and the host bone, and the bone grew deeper in the composite aerogel scaffold group with SOST monoclonal antibody + SDF-1. To further confirm that the SOST monoclonal antibody + SDF-1 composite aerogel scaffold has better osteogenic and angiogenic effects, we removed tissues from the target area to detect the osteogenic and angiogenic genes ALP, BMP2, and VEGF by fluorescence quantitative PCR and Western blot. The results showed that compared with the control and the scaffold with a single drug, the composite aerogel scaffold with SOST monoclonal antibody + SDF-1 had more advantages in osteogenesis and angiogenesis.

## Conclusion

In this study, an NCF-Col-NHA aerogel scaffold was successfully prepared, which has a three-dimensional porous structure, excellent anti-deformation ability, and good biomechanical properties, and successfully loaded with SOST monoclonal antibody and SDF-1, which can promote osteogenesis and angiogenesis and achieve sustained and effective drug release. The scaffold showed good osteogenic and angiogenic effects *in vitro* and *in vivo*. Tissue repair can be realized in the osteonecrosis area of steroid-induced ONFH. These results indicate that the NCF-Col-NHA aerogel scaffold with SOST McAb + SDF-1 is a promising substitute for bone repair.

## Data Availability

The original contributions presented in the study are included in the article/Supplementary Material, further inquiries can be directed to the corresponding author.
