# Supplementary material for: In vitro and in vivo Repair Effects of the NCF-Col-NHA Aerogel Scaffold Loaded With SOST Monoclonal Antibody and SDF-1 in Steroid-Induced Osteonecrosis
Source: Front Bioeng Biotechnol. 2022 Mar 8;10:825231. doi: 10.3389/fbioe.2022.825231 (PMC8964358; doi:10.3389/fbioe.2022.825231)
Supplement: Supplementary file 1 [file DataSheet1.docx]

**In vitro and in vivo repair effects of the NCF-Col-NHA aerogel scaffold loaded with SOST monoclonal antibody and SDF-1 in steroid-induced osteonecrosis**

**Supporting information**

**Table S1. List of supplies used for the current study**

| **Supplies** | **Manufacturer** | **Catalog Number** |
| --- | --- | --- |
| SDF-1 monoclonal antibody | ORIGENE (USA) | AP20632PU-N |
| SOST monoclonal antibody | ORIGENE (USA) | AP13236PU-N |
| Nanohydroxyapatite | Sigma (USA) | 677418 |
| Lignocellulose | Qi-hong Science and Technology (China) | 11132-73-3 |
| Type I collagen | Bo-Mei Biological (China) | 9007-34-5 |
| DMEM medium | Gibco (USA) | 30030 |
| Fetal Bovine Serum | Gibco (USA) | 10099141C |
| Human Vascular Endothelial cell line | iCellBioscience Inc. (China) | iCell-H110 |
| PCR Primers | Seville Biotechnology Co. | GM2001 |

SDF-1, stromal cell-derived factor-1; SOST, sclerostin; DMEM, Dulbecco’s modified Eagle’s medium; MSC, mesenchymal stem cell; PCR, polymerase chain reaction

**Table S2. Primers used for real-time RT–PCR**

| **Gene** | **Forward (5’-3’)** | **Reverse (5’-3’)** |
| --- | --- | --- |
| *GAPDH* | *TGAAGGTCGGAGTGAACGGAT* | *CGTTCTCAGCCTTGACCGTG* |
| *ALP* | *GAGCGACACGGACAAGAAACC* | *CATCACATGAGGGATGTAGTTCTGC* |
| *VEGF* | *AGGCGAGGCAGCTTGAGTTA* | *CAAGGACTGTTCTGTCGGTGG* |
| *BMP-2* | *GTGAATCAGAACACGAGCCG* | *CAAAGACCTGCTAATCCTCACG* |
